# Targeted Physical Function Exercises for Frailty and Falls Management in Pre-Frail Community-Dwelling Older Adults: A Randomized Controlled Trial

**DOI:** 10.3390/healthcare13192486

**Published:** 2025-09-30

**Authors:** Ioannis Savvakis, Athina Patelarou, Enkeleint A. Mechili, Eirini Stratidaki, Evridiki Patelarou, Konstantinos Giakoumidakis

**Affiliations:** 1 Department of Nursing, School of Health Sciences, Hellenic Mediterranean University, 71410 Heraklion, Greece; 2 Laboratory of Evidence-Based Healthcare, Education and Clinical Protocols, Department of Nursing, School of Health Sciences, Hellenic Mediterranean University, 71410 Heraklion, Greece; 3 Department of Healthcare, Faculty of Health, University of Vlora, 9401 Vlora, Albania

**Keywords:** physical function exercise, frailty, concern about falling, older adults

## Abstract

**Background/Objectives:** Exercise is essential for older adults to maintain or improve their physical condition. This study aimed to investigate whether improvements in physical performance, functional mobility, and balance through targeted physical function exercises could positively influence Concerns about Falling (CaF) and frailty in pre-frail community-dwelling older adults. **Methods:** We conducted an 18-month randomized controlled trial involving 112 pre-frail community-dwelling older adults aged 65 years or older. 55 individuals in the control group (CG) and 57 in the intervention group (IG) were assessed. The IG participated in a home-based physical function exercise program. Primary outcomes included Physical Performance (Short Physical Performance Battery, SPPB), Functional Mobility (Timed Up and Go, TUG), Balance (Berg Balance Scale, BBS), CaF (Falls Efficacy Scale–International, FES-I), and Frailty status (SHARE-FI). Assessments were conducted at baseline, 6, 12, and 18 months. **Results:** The IG showed significant improvements in BBS (*p* < 0.01, partial eta^2^ 0.17), SPPB (*p* < 0.01, partial eta^2^ 0.13), TUG (*p* < 0.01, partial eta^2^ 0.14) and FES-I (*p* < 0.01, partial eta^2^ 0.07) compared to the CG and their baseline after 6, 12 and 18 months of intervention. By 18 months, frailty status improved in the IG, with 12.3% classified as non-frail compared to 2.0% in the CG, while 14.5% of the CG transitioned to frailty versus none in the IG. **Discussion:** The intervention appears to support improvements in physical function and may contribute to reductions in CaF and beneficial changes in frailty status among pre-frail community-dwelling older adults.

## 1. Introduction

Frailty is a condition characterized by reduced mobility, strength, and physical function that affects the quality of life of older adults by increasing their vulnerability to falls and disability [1]. In addition to physical decline, frailty can have a negative emotional and social impact, affecting older adults’ independence and healthy active aging [2]. Frailty is increasingly recognized as a dynamic condition with potential for improvement, particularly in its early stages [3]. This understanding underscores the importance of timely intervention to prevent further deterioration and reduce the risk of adverse outcomes.

Concerns about Falling (CaF) is a feeling of worry about situations that may threaten or challenge an individual’s balance, increasing the risk of falls with negative effects on the physical, social, and psychological state of older adults [4]. CaF is associated with a limitation of daily functional activities and, by extension, with a reduction in strength and balance control, leading to a vicious cycle of reduced physical activity participation, ultimately accelerating the process of physical frailty [5].

Exercise interventions that aim to increase strength, balance, coordination, and mobility have a positive impact on reducing falls and fractures due to falls [6] and appear to be effective in reducing CaF and managing frailty [7]. Implementing appropriate intervention programs, frail and pre-frail older adults can enhance their physical function and reduce the development of fall-related concerns [8].

Additionally, exercise interventions contribute to physical resilience by increasing walking speed and improving grip strength, key indicators of frailty severity [9]. Evidence from recent randomized controlled trials indicates that multicomponent exercise programs, including those delivered through primary care or combined with home-based approaches, can significantly improve physical performance and contribute to the reversal of frailty in older adults [10,11].

However, physical function exercises are a dynamic process that can evolve. Targeted physical function exercises are structured interventions designed to address specific deficits in physical abilities, such as balance, strength, and mobility, to improve functional performance in daily activities. In contrast to general exercise programs, these exercises are individualized in intensity and difficulty, and are task-specific, focusing on real-life movements to restore or maintain functional independence [12].

Despite growing evidence, long-term interventions with extended follow-up remain underrepresented in research involving frail and pre-frail older adults [13]. This study aims to contribute to the existing evidence by providing recent data on the sustained impact of structured exercise in this population.

The objective of this study was to evaluate the effectiveness of tailored interventions enhancing physical performance, functional balance, and mobility on the management of frailty and CaF in pre-frail community-dwelling older adults. We hypothesized that such interventions would lead to significant improvements in frailty status and CaF, in this population. Ultimately, the aim was to empower pre-frail older adults to prevent fitness decline, maintain independence, and enhance their healthy aging.

## 2. Methods

### 2.1. Trial Design

The present study was an 18-month randomized controlled trial (RCT) involving pre-frail participants, with one intervention group (IG) and one control group (CG). Baseline measurements were followed by assessments at 6 months (T1), 12 months (T2), and 18 months (T3). Participant recruitment began in September 2022, with interventions starting in December 2022 and concluding in June 2024.

### 2.2. Participants

Eligible participants were 65 years and older, classified as pre-frail according to the SHARE-Frailty Instrument [14], and were physically independent in walking and performing daily activities. Participants classified as frail or non-frail were excluded. The exclusion criteria included severe contraindications for physical exercise, central nervous system impairments, inability to speak and understand Greek, psychiatric disorders, and a cognitive state that prevents understanding the researcher’s instructions. All conditions were determined based on diagnoses documented by the participants’ physicians.

**Figure 1:**
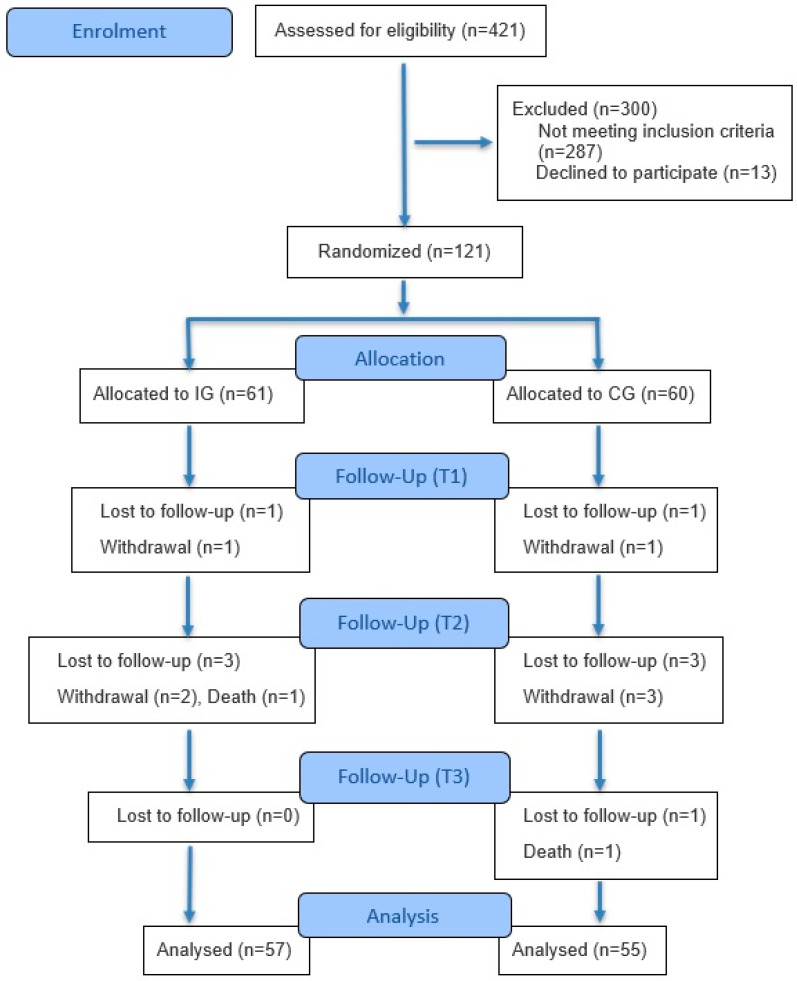
CONSORT flow diagram illustrating each stage of the trial.

A total of 421 participants were screened. Among them, 300 were excluded due to different reasons. Six met the exclusion criteria (1 had exercise contraindications from the cardiologist due to recent surgery, 1 had Parkinson’s disease, 1 had multiple sclerosis, 2 had a recent severe stroke without being able to move, and 1 did not speak the Greek language), and 13 declined to participate. An additional 281 participants were excluded, with 227 classified as non-frail and 54 as frail. 121 Pre-frail participants were randomly assigned to an IG (*n* = 61) and a CG (*n* = 60), using a computerized randomization sequence. As shown in Figure 1, at the end of the intervention period at 18 months of follow-up, 55 individuals in the CG and 57 in the IG were assessed.

### 2.3. Sample

This pragmatic randomized controlled trial was conducted with a sample size determined by feasibility and the availability of eligible participants in the target population. A total of 112 pre-frail community-dwelling older adults were assessed.

### 2.4. Procedure

All individuals were recruited via telephone contact and through referral to a single Primary Medical Center of the Municipality of Archanes-Asterousia in the wider area of Heraklion, Crete, for their assessment, were thoroughly informed about the study, and provided written informed consent before participation. Participants then completed a form with basic sociodemographic information and underwent all baseline assessments. All database and personal information were collected solely for the study, with access and responsibility for confidentiality resting exclusively with the principal researcher. One researcher created a computerized randomization sequence, and the participants were randomized (1:1 ratio) to the IG and the CG, creating two folders with the participants of each group. A blinded researcher opened the folders of the allocation and assigned each participant to their group. The trial could not be blinded as the content and objectives of the exercise sessions were obvious to both participants and researchers. Although outcome assessors were initially blinded to group allocation, blinding could not be maintained in practice, as participants often disclosed their participation status during follow-up assessments.

### 2.5. Intervention

The intervention was carried out by the principal researcher, a physiotherapist, who prescribed a home-based exercise program tailored to each participant in the IG according to their functional ability. The first meeting was held at the Medical Center, where the physiotherapist explained the importance of the intervention and addressed any questions. Each participant received individualized instruction and a demonstration of the exercises. Initial intensity was determined based on the participant’s performance and tolerance during the demonstration session, considering factors such as balance, mobility, strength, and perceived exertion.

Participants were instructed to complete three sessions per week, on days of their choice, each lasting 30–35 min. Each participant received an exercise diary and a guide with illustrated instructions, including the recommended number of repetitions and frequency. Exercises were prescribed with an initial target of three sets of ten repetitions per exercise. If this was too challenging, the volume was adjusted (e.g., six sets of five repetitions), and the duration of each exercise was kept within 1 to 1.5 min, with a 30-s rest period between exercises.

Progression was incorporated throughout the 18-month program by increasing exercise complexity, or repetitions, depending on the participant’s progress and feedback. The physiotherapist monitored progress through monthly follow-up phone calls, during which participants could also request modifications or clarifications to ensure that exercise remained safe, challenging, and achievable.

Every session started with a 5-min walk as a whole-body warm-up. Physical function interventions involved progressively increasing intensity strength exercises, beginning with or without an elastic resistance band. The resistance level varied according to the stiffness of the band, progressing in the following order: yellow, green, and black. The yellow band provided the least resistance, while the black band offered the greatest. These exercises targeted the following muscle groups: the hip abductors and extensors, knee extensors, shoulder abductors, and external rotators. Other exercises consisted of standing calf raises, squats, bridges, and push-ups. Additionally, functional exercises included heel-to-toe standing or walking, single-leg standing, stepping over obstacles, bending down and lifting an object (Table 1). At the end of each session, participants are encouraged to spend 5 min stretching to enhance their range of motion and improve joint mobility.

Participants in the control group were instructed to continue their usual daily activities and routines without any structured intervention. They were not restricted from engaging in physical activity or other personal habits, but no exercise guidance or health program was provided. To maintain participant engagement and balance researcher–participant contact between groups, control group members received a monthly social telephone call. Every six months from the beginning of the interventions, a follow-up assessment was scheduled (T1, T2, T3) until the end of the trial, 18 months later, for all participants in both groups.

### 2.6. Outcomes

Information about age, gender, BMI, marital status, profession, smoking habit, homebound status, number of children, and living arrangements (living alone or with others) was obtained at baseline measurements. Assessments were conducted at baseline, 6 months (T1), 12 months (T2), and at the end of the intervention period at 18 months (T3). All questionnaires were completed by the researchers based on participants’ responses and through observation of the activities they were asked to perform. The physiotherapist who led the intervention was not involved in any assessment.

### 2.7. Primary Outcome Measures

The level of frailty was assessed by using the SHARE-Frailty Instrument [14]. It is a valid and brief tool for screening and monitoring frailty in European (including the Greek population) community-dwelling adults aged ≥50, given five quick and simple measurements [14], related to Fried’s criteria (involuntary loss of weight, exhaustion, low activity, slowness, and weakness) [15], determining the category of frailty (frail, pre-frail, non-frail) [14].

The Short Physical Performance Battery (SPPB) [16] was used to assess physical performance. It is a valid tool consisting of 3 tests in three different domains (walking, sit-to-stand, and balance) to assess function. Each test is rated on a scale of 0 to 4, summed to give an overall score ranging from 0 to 12, with the higher score indicating better performance [16]. No specialized equipment is required other than a stopwatch and an armchair. The intraclass correlation coefficient (ICC) for SPPB was 0.953, indicating high reliability.

For quantifying functional mobility, the Timed Up and Go Test (TUG) was used. It is a valid, quick test that requires no special equipment or training. The participants were observed and timed while standing up from an armchair, walking three meters, making a turn, and returning to their sitting position. The timer started when the participants were ready to stand up and stopped when they returned to sit [17]. The reliability of this test was excellent (ICC = 0.986).

The Berg Balance Scale (BBS) was used to assess a participant’s ability to balance safely during specific tasks. It evaluates dynamic and static balance through 14 functional tasks, with each task answered on a five-point scale ranging from 0, indicating the lowest level of functioning, to 4, the highest level of functioning. The final summary score ranges from 0 to 56 [18]. Psychometric testing of this scale revealed validity and reliability in Greek clinical settings [19]. It has shown good reliability (ICC = 0.888).

The Falls Efficacy Scale International (FES-I) is a 16-item questionnaire that assesses the participant’s fall efficacy, measuring the level of CaF during various social and physical activities. Every item has a four-point (1–4) score, summarizing a total score of 64 points. 16–22 indicates low concern about falling, and 23–64 high concern [20]. It is a valid and reliable scale for Greek community-dwelling older adults [21]. The reliability of this scale was excellent (ICC = 0.993).

### 2.8. Secondary Outcome Measures

To assess the quality of life of the participants, the Greek version of the WHOQOL-BREF scale was used [22], which includes 26 items concerning 4 domains of health (physical health, psychological health, social relationships, and environment). It also contains overall quality of life and general health items. Each item of the WHOQOL-BREF is scored from 1 to 5 on a response scale, which is stipulated as a five-point ordinal scale. The scores are then transformed linearly to a 0–100 scale [23]. The ICC for this scale was 0.840, indicating good reliability for each measure.

The short-form Geriatric Depression Scale (GDS), consisting of 15 yes-or-no questions with a total score range of 0 to 15, was used to assess the participants’ depressive symptoms. A score of up to 5 indicates no depression, 6–10 mild depression, and 11–15 severe depression [24]. It is a valid scale for the Greek population [25]. The reliability of this scale was excellent (ICC = 0.943).

### 2.9. Ethical Consideration

This study was conducted according to the CONSORT 2025 recommendations for clinical trials [26] and followed the Declaration of Helsinki ethical principles. The research protocol was approved by the Research Ethics Committee of the Hellenic Mediterranean University (2021.46) and the Social Solidarity Organization of the Municipality (2021.60). This trial was registered retrospectively at the Clinical Trials Register (ID number NCT06731712, Date of registration: 26 October 2024).

### 2.10. Statistical Analysis

The Shapiro–Wilk test was used to check normality. Values were expressed as mean Standard Deviation (SD) for continuous data, and frequencies were presented in the case of categorical variables. A bar chart was used for frailty to check the distribution at the four time points. A two-way mixed-model analysis of variance (ANOVA) was conducted to determine within-group, between-group, and group × time interaction effects. In addition to reporting *p*-values, effect sizes were calculated to provide an estimate of the magnitude of the observed effects. We report partial eta^2^, as it is the recommended measure of effect size in ANOVA models since it reflects the proportion of total variance explained by each factor after accounting for other effects in the model. Conventional benchmarks were used for interpretation: small (partial eta^2^ = 0.01), medium (partial eta^2^ = 0.06), and large (partial eta^2^ = 0.14) Tukey’s post hoc procedures were performed to locate the pairwise differences between the mean values. Data analysis was carried out using Stata 16.0 (StataCorp, College Station, TX, USA) and IBM SPSS Statistics for Windows, Version 26 (Released 2019; IBM Corp., Armonk, NY, USA). No missing data was observed during analysis.

## 3. Results

The baseline characteristics of the 112 participants are presented in Table 2. Of these, 57 were randomly allocated to the intervention group and 55 to the control group. The mean age of the total sample was 79.3 years (SD = 5.5), and the majority were female (72.3%). Approximately 29.5% of participants reported living alone.

The intervention and control groups were generally comparable in terms of key sociodemographic and clinical characteristics. The IG had a slightly lower mean age (78.7 ± 5.8 years) compared to the CG (79.9 ± 5.2 years), and a somewhat higher proportion of female participants (75.4% vs. 69.1%). Small differences were also noted in marital status and homebound classification, with a greater proportion of married and non-confined individuals in the IG. A higher percentage of participants in the CG reported a history of smoking.

**Figure 2:**
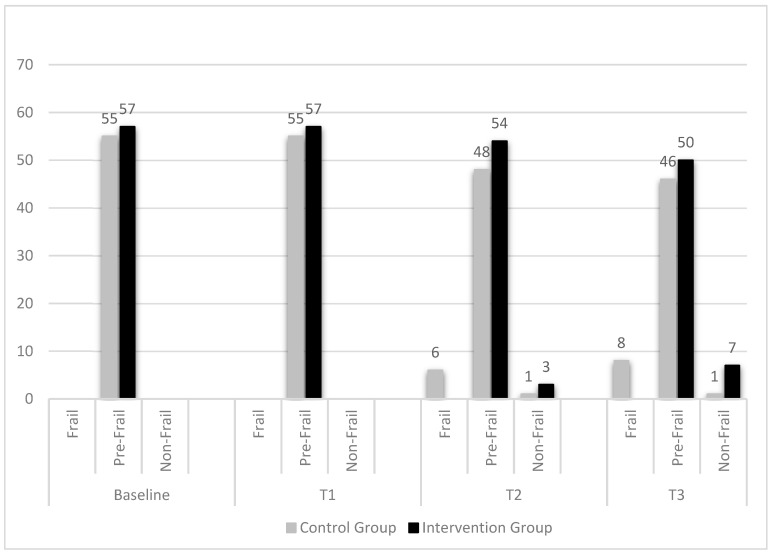
Distribution of frailty status by group and time points.

Baseline and post-intervention (6, 12, and 18 months) primary outcome data are presented in Figure 2 and Table 3. As shown in Figure 2, the distribution of frailty status began to diverge between the groups at 12 months (T2) and became more pronounced at 18 months (T3). At T2, 3 participants in the IG transitioned to a non-frail status, whereas in the CG, 6 participants progressed to frailty, and 1 improved to non-frail. By T3, 7 participants in the IG were classified as non-frail. In contrast, 8 participants in the CG transitioned to frailty, while just 1 maintained the non-frail status. The probability of being classified as non-frail versus pre-frail at T3 was 12.3% in the IG, compared to 2.0% in the CG. Conversely, the probability of being classified as frail versus pre-frail at T3 was 0% in the IG and 14.5% in the CG, suggesting a favorable shift in frailty status among participants who received the targeted exercise intervention.

Mixed model repeated measure ANOVA demonstrated significant group x time interaction effects for physical performance (SPPB scale) (*p* < 0.001, partial eta^2^ 0.13), balance (BBS) (*p* < 0.001, partial eta^2^ 0.17), mobility (TUG test) (*p* < 0.001, partial eta^2^ 0.14), and CaF (FES-I) (*p* < 0.001, partial eta^2^ 0.07). According to conventional thresholds, these effects range from medium to large in magnitude. As shown in Table 3, post hoc analyses revealed that the intervention group demonstrated statistically significant improvements within-group in all these outcomes at all follow-up points (*p* < 0.05). In contrast, the control group exhibited significant declines over time in SPPB, BBS, and TUG scores (*p* < 0.001), while FES-I scores remained largely unchanged. Between-group comparisons revealed significantly greater improvements in the intervention group at 12 and 18 months for all outcomes (*p* < 0.05), with earlier differences emerging for FES-I at the 6-month follow-up.

ANOVA also revealed a significant group × time interaction for depressive symptoms (GDS) (*p* < 0.001, partial eta^2^ 0.09), while interaction effects for the WHOQOL-BREF scale were significant only in certain domains (Table 4). By 18 months, participants in the IG had a significantly greater reduction in GDS score compared to controls (*p* = 0.029). Within-group analyses showed that the IG experienced a significant reduction in GDS scores from baseline across all follow-up points (*p* < 0.001). Additionally, the IG registered significant improvements in most of the WHOQOL-BREF scale domains compared to baseline at 6, 12, and 18 months.

## 4. Discussion

The study results showed that a targeted physical function exercise intervention can enhance physical performance, functional mobility, and balance in pre-frail older adults living in the community. These findings confirm the study hypothesis, demonstrating significant improvements with a positive impact on reducing CaF and managing frailty. Our study intervention plan complies with the World Health Organization recommendations for older adults aged 65 years and above. In order to enhance functional capacity and prevent falls, it is recommended that older adults perform varied multicomponent activity 3 or more times per week [27]. The intervention included home-based exercises. Only three participants from the intervention group dropped out, which may reflect the program’s potential feasibility and acceptability. In addition, what may have favored their participation were the guidelines given before and during the intervention.

The partial eta^2^ values observed for key outcomes suggest that the intervention effects were not only statistically significant but also clinically meaningful. Specifically, balance (BBS) showed a large effect size (partial eta^2^ = 0.17), indicating substantial improvements that are directly relevant for reducing fall risk and enhancing stability in daily activities. Functional mobility (TUG) and physical performance (SPPB) demonstrated moderate-to-large effects (partial eta^2^ = 0.14 and 0.13, respectively), reflecting consistent gains across time that may translate into greater independence in walking, transferring, and performing routine tasks. Although the reduction in CaF (FES-I) showed a medium effect size (partial eta^2^ = 0.07), it nonetheless points to a meaningful psychological benefit, as even moderate reductions in fear of falling can improve confidence and willingness to engage in physical and social activities. Collectively, these effect sizes underscore the clinical relevance of the intervention in improving both physical capacity and psychosocial outcomes among pre-frail older adults, with potential implications for fall prevention and maintenance of autonomy.

Our findings are in line with recent studies indicating that structured exercise programs for older adults can improve physical performance, mobility, and balance while reducing frailty and fall risk [11,28]. Interventions involving exercises improving strength, mobility, and balance in older adults also address the fear of falling and overall frailty [8,29]. These programs are often associated with improved quality of life, highlighting their importance for this population [29].

It is widely recognized that resistance and balance exercises are essential for increasing muscle mass and improving muscle strength, mobility, and balance in older adults [30,31]. Our findings align with recent studies involving resistance, mobility, and balance exercises, including resistance exercises with elastic bands, which can have a positive effect on physical function in pre-frail older adults [32,33].

However, unlike previous studies that focused on institutionalized older adults [30,34] or those with severe frailty [35,36,37], our study targeted a pre-frail population living independently in the community. By addressing this population, our findings contribute to the body of evidence supporting tailored exercise programs aimed at maintaining independence and preventing frailty progression. Moreover, a home-based intervention in a community-dwelling population may offer insights into the feasibility and relevance of such programs in everyday settings, beyond clinical or institutional environments [11,28].

Although the IG demonstrated a statistically significant improvement in SPPB scores (mean = 8.2) compared to the CG (mean = 7.6), this change did not reach the minimum clinically important difference (MCID) of 1 point [38] (mean difference = 0.6, *p* = 0.002). This suggests that, while the intervention had a measurable effect, the magnitude of improvement may not have been sufficient to result in noticeable functional benefits in everyday life. Therefore, the clinical relevance of this finding should be interpreted with caution.

Fall efficacy is related to an individual’s perceived ability to perform activities without falling. The FES-I assessment tool focuses on an individual’s fall efficacy by assessing the level of CaF during social and physical activities [21]. Higher FES-I scores indicate greater concerns about falling, often linked to reduced activity and loss of independence, whereas lower scores reflect greater confidence in daily activities. In our study, it appears that increasing the physical function of pre-frail older adults reduces their CaF, leading to lower FES-I scores and thus greater ability to perform activities without falling. This suggests a clinically meaningful improvement in fall-related self-efficacy among pre-frail older adults. These results agree with a recent study showing that exercises enhancing strength, balance, and mobility improve fall efficacy in older adults [39].

Although baseline values were somewhat lower in the control group (Table 4), no statistically significant differences were detected between-groups, and these imbalances were interpreted as chance variation in the context of a randomized sample with modest size. We found a positive effect of physical function interventions on the physical health domain of quality of life, in the first 6 months of follow-up (*p* = 0.007) between the two groups, which effect does not seem to be maintained until the 18th month of follow-up, although there is a statistically significant difference (*p* = 0.049). This may be attributed to the participants’ initial enthusiasm, willingness, and greater commitment to performing the exercises during the first 6 months. Following the first follow-up, their level of physical activity may have declined during the summer period, and they may not have maintained the same dedication and intensity in the exercise program. Additionally, it is possible that the participants may have become accustomed to their physical improvements and no longer perceived them as significant, even if they were still present.

Similarly, in the environmental domain, at the 18th month of follow-up, there seems to be a statistically significant difference between the two groups (*p* = 0.030). This domain reflects how satisfied and safe people feel with their environment and with their access to various services. Regarding the psychological health domain, there is a positive effect of the interventions and a statistically significant difference between groups (*p* = 0.040). Along with the improvement in the psychological health domain, the occurrence of depressive symptoms decreased significantly (*p* = 0.029). In the present study, the changes in these domains of quality of life and depression are likely to be directly related to the positive improvements of the intervention in physical function, in line with previous studies [40,41]. It is well known that exercise increases brain-derived neurotrophic factor (BDNF) levels, a protein involved in neuroplasticity and neurogenesis, particularly in the hippocampus, a brain region involved in mood regulation. Moreover, higher BDNF levels modulate neurotransmitter systems, including serotonin and dopamine pathways, which contribute to improved emotional well-being and reduced depressive symptoms [42,43]. What is noteworthy is that in both the intervention and control groups, the psychological health domain and depression show improvement within groups by time points. This could be explained by the long duration of the study and its initiation during the emotionally difficult period for the population, just after the COVID-19 (SARS-CoV-2) pandemic restrictions. Therefore, it is expected that there will be an improvement in the psychological health of the population over time.

Frailty status appeared to improve over time among participants in the IG. By the 18th month of follow-up, seven individuals in the IG had transitioned from pre-frailty to non-frailty status. In contrast, the CG showed a less favorable trend: eight participants progressed from pre-frailty to frailty during the same period. These transitions suggest that home-based physical function interventions may help reverse early stages of frailty and support a return to functional independence. Conversely, the absence of structured intervention in the CG highlights the potential for deterioration in frailty status over time without early preventive strategies. These findings reinforce the importance of timely, accessible programs aimed at maintaining and improving physical resilience in community-dwelling older adults. However, the predominantly female sample and recruitment from a single geographic region should be considered when interpreting the generalizability of these findings.

## 5. Limitation

A key limitation of the study is the lack of blinding, since neither participants nor assessors could be blinded; this may have introduced some degree of assessment bias. This methodological issue is explicitly acknowledged and should be carefully considered when interpreting the study findings. The intervention was not conducted in a community room under the supervision of the researcher, which may have affected the overall results. The sample size in this study was based on feasibility and participant availability rather than on a formal a priori power calculation. The absence of an active CG may have introduced bias, as participants in this group did not receive a structured intervention, which could have affected their engagement or outcomes. The assessment of various outcomes was performed through self-reporting by the participants. This may raise concerns about the accuracy of the answers given. As adherence data recorded in participant diaries were not systematically analyzed, compliance with the exercise protocol (e.g., participation rates) could not be reported. This limitation prevents a full assessment of intervention feasibility and should be addressed in future studies. The lack of mechanistic data on how intervention affects biological processes associated with aging is another limitation. Diet, rest time, and medication adherence were not recorded in this study. These variables may potentially have an impact on the results.

## 6. Conclusions

The results indicated that targeted physical function exercises can enhance physical performance, functional mobility, and balance while limiting CaF among pre-frail community-dwelling older adults. Additionally, the intervention was associated with better management of frailty status. These findings suggest that incorporating targeted physical function exercises into an intervention plan is a feasible and effective approach for falls and frailty management in this population. Future studies may benefit from incorporating an active control group to reduce potential bias associated with differential attention or participant expectations. Furthermore, they should investigate the biological mechanisms underlying the intervention, such as biomarkers or molecular changes associated with aging and frailty.

## Figures and Tables

**Table 1:** Physical function exercises protocol.

Exercises	Beginner	Intermediate	Advanced
Hip abduction & extension	Standing with or without a light resistance band	Standing with a medium resistance band	Standing with a heavy resistance band
Knee extension	Seated with or without a light resistance band	Seated with a medium resistance band	Seated with a heavy resistance band
Shoulder abduction & external rotation	Standing with or without a light resistance band	Standing with a medium resistance band	Standing with a heavy resistance band
Standing calf raises	Holding onto a stable surface	Hands-free, slow, and controlled movements	Single-leg calf raises
Squats	Chair-assisted squats	Bodyweight squats	Weighted squats (holding an object)
Bridges	Assisted with arms pushing off the floor	Standard glute bridge	Single-leg bridge
Push-ups	Wall push-ups	Table push-ups	Standard or knee push-ups
Heel-to-toe standing/walking	Holding onto a stable surface	Hands-free standing	Walking heel-to-toe with a controlled pace
Single-leg standing	Holding onto support	Hands-free, eyes open	Hands-free, eyes closed
Stepping over obstacles	Small height obstacles	Moderate-height obstacles	Higher obstacles
Bending down—Lifting an object	Using a chair for support—Lightweight object	Hands-free, slow movement—Medium-weight object	Hands-free with feet together—Heavier object

**Table 2:** Baseline characteristics of study participants.

Characteristics	Total (*n* = 112)	Control Group (*n* = 55)	Intervention Group (*n* = 57)
**Age** (years), *mean (SD)*	79.3 (5.5)	79.9 (5.2)	78.7 (5.8)
**Sex,** *n* (%)			
Male	31 (27.7)	17 (30.9)	14 (24.6)
Female	81 (72.3)	38 (69.1)	43 (75.4)
**Marital status,** *n* (%)			
Married	77 (68.8)	36 (65.5)	41 (71.9)
Widowed/divorced	35 (31.3)	19 (34.5)	16 (28.1)
**Ever smoked,** *n* (%)			
Yes	81 (72.3)	44 (80.0)	37 (64.9)
No	31 (27.7)	11 (20.0)	20 (35.1)
**Profession,** *n* (%)			
Intellectual work	10 (8.9)	6 (10.9)	4 (7.0)
Manual work	96 (85.7)	46 (83.6)	50 (87.7)
Combination of manual and practical work	6 (5.4)	3 (5.5)	3 (5.3)
**Number of children,** *mean (SD)*	1.9 (0.9)	1.9 (0.9)	1.9 (0.8)
**Live alone,** *n* (%)			
Yes	33 (29.5)	19 (34.5)	14 (24.6)
No	79 (70.5)	36 (65.5)	43 (75.4)
**BMI** (kg/cm^2^), *mean (SD)*	25.7 (1.8)	26.0 (2.1)	25.5 (1.3)
**Homebound,** *n* (%)			
Semi-confined	53 (47.3)	30 (54.5)	23 (40.3)
Non-confined	59 (52.7)	25 (45.5)	34 (59.6)

**Table 3:** Mean Baseline Scores and Mean Change (Δ = Follow-up − Baseline) in Physical Performance, Balance, Functional Mobility, and CaF at 6, 12, and 18 Months by Group.

	Baseline	Δ-T1 (6 Months)	Δ-T2 (12 Months)	Δ-T3 (18 Months)	*p*-Value Within Group
**SPPB**					**<0.001 ^$^**
Intervention Group	8.0 ± 0.8	0.05 ± 0.35	0.16 ± 0.49	0.16 ± 0.49	0.012 ^#^
Control Group	8.0 ± 0.7	−0.11 ± 0.31	−0.31 ± 0.57	−0.42 ± 0.63	**<0.001 ^#^**
***p*** **-value between Groups**	0.721	0.205	0.013 *	0.002 *	
**BBS**					**<0.001 ^$^**
Intervention Group	48.1 ± 1.8	0.49 ± 1.34	0.75 ± 1.76	0.68 ± 1.97	**<0.001 ^#^**
Control Group	48.5 ± 1.8	−0.96 ± 1.91	−1.49 ± 2.88	−2.16 ± 3.29	**<0.001 ^#^**
***p*** **-value between Groups**	0.283	0.019 *	0.002 *	<0.001 *	
**TUG**					**<0.001 ^$^**
Intervention Group	9.5 ± 1.3	−0.2 ± 0.45	−0.12 ± 0.55	0.05 ± 0.47	**<0.001 ^#^**
Control Group	9.8 ± 1.8	0.42 ± 0.55	0.52 ± 0.63	0.6 ± 0.79	**<0.001 ^#^**
***p*** **-value between Groups**	0.677	0.009 *	0.012 *	0.045 *	
**FES-I**					**<0.001 ^$^**
Intervention Group	19.5 ± 2.7	−0.37 ± 0.64	−0.46 ± 0.95	−0.37 ± 0.94	**<0.001 ^#^**
Control Group	20.1 ± 2.8	0.05 ± 0.23	0.05 ± 0.36	0.05 ± 0.59	0.733
***p*** **-value between Groups**	0.225	0.031 *	0.019 *	0.043 *	

Values are mean ± SD; Change values (Δ) reflect the difference from baseline at each time point; Within-group *p*-values represent changes over time; between-group *p*-values compare intervention and control groups at each time point. SPPB, Short Physical Performance Battery; BBS, Berg Balance Scale; TUG, Timed Up and Go Test; FES-I, Falls Efficacy Scale-International. * Statistically significant differences at *p* value < 0.05 between groups by time points. ^#^ Statistically significant differences at *p* value < 0.05 within groups by time points. ^$^ Statistically significant differences at *p* value < 0.05 for the interaction term between groups and time points. Bold indications correspond to statistically significant findings.

**Table 4:** Mean Baseline Scores and Mean Change (Δ = Follow-up − Baseline) in Quality of Life and Depressive Symptoms at 6, 12, and 18 Months by Group.

	Baseline	Δ-T1 (6 Months)	Δ-T2 (12 Months)	Δ-T3 (18 Months)	*p*-Value Within Group
**Physical Health**					0.504
Intervention Group	45.6 ± 13.7	3.26 ± 10.04	3.38 ± 10.2	2.19 ± 9.59	**0.002 ^#^**
Control Group	41.7 ± 10.1	2.27 ± 10.11	2.21 ± 10.14	2.4 ± 9.57	0.055
***p*** **-value between Groups**	0.218	0.007 *	0.005 *	0.049 *	
**Psychological Health**					0.440
Intervention Group	45.8 ± 14.3	2.12 ± 5.85	2.27 ± 5.95	3.22 ± 7.64	**<0.001 ^#^**
Control Group	41.1 ± 12.1	3.48 ± 6.45	3.48 ± 6.89	3.71 ± 7.97	**<0.001 ^#^**
***p*** **-value between Groups**	0.071	0.093	0.082	0.040 *	
**Social Relationships**					0.704
Intervention Group	41.7 ± 15.1	0	0.29 ± 1.55	0.15 ± 1.92	0.532
Control Group	40.3 ± 13.2	0.15 ± 1.96	0.15 ± 1.96	0 ± 1.6	0.881
***p*** **-value between Groups**	0.550	0.613	0.532	0.488	
**Environment**					0.056
Intervention Group	44.8 ± 12.5	0.05 ± 1.62	0.16 ± 1.91	1.48 ± 4.34	**0.010 ^#^**
Control Group	41.4 ± 10.9	−0.06 ± 1.12	−0.28 ± 1.24	−0.17 ± 3.15	0.330
***p*** **-value between Groups**	0.129	0.101	0.064	0.030 *	
**Overall Quality of Life and General Health**					0.156
Intervention Group	44.3 ± 17.7	1.1 ± 3.57	0.88 ± 3.22	2.41 ± 6.44	**0.024 ^#^**
Control Group	42.5 ± 15.3	0.23 ± 2.94	0 ± 3.4	0 ± 5.89	0.940
***p*** **-value between Groups**	0.881	0.669	0.660	0.301	
**GDS**					**<0.001 ^$^**
Intervention Group	5.23 ± 1.5	−0.4 ± 0.53	−0.61 ± 0.62	−0.93 ± 0.88	**<0.001 ^#^**
Control Group	5.07 ± 1.4	−0.31 ± 0.57	−0.29 ± 0.63	−0.36 ± 0.65	**<0.001 ^#^**
***p*** **-value between Groups**	0.584	0.814	0.478	0.029 *	

Values are mean ± SD; Change values (Δ) reflect the difference from baseline at each time point; Within-group *p*-values represent changes over time; between-group *p*-values compare intervention and control groups at each time point. GDS, Geriatric Depression Scale. * Statistically significant differences at *p* value < 0.05 between groups by time points. ^#^ Statistically significant differences at *p* value < 0.05 within groups by time points. ^$^ Statistically significant differences at *p* value < 0.05 for the interaction term between groups and time points. Bold indications correspond to statistically significant findings.

## Data Availability

The data presented in this study are available on request from the corresponding author.
